# Fourier Transform Infrared (FTIR) Spectroscopic Study of Biofilms Formed by the Rhizobacterium *Azospirillum baldaniorum* Sp245: Aspects of Methodology and Matrix Composition

**DOI:** 10.3390/molecules28041949

**Published:** 2023-02-18

**Authors:** Alexander A. Kamnev, Yulia A. Dyatlova, Odissey A. Kenzhegulov, Yulia P. Fedonenko, Stella S. Evstigneeva, Anna V. Tugarova

**Affiliations:** Laboratory of Biochemistry, Institute of Biochemistry and Physiology of Plants and Microorganisms—Subdivision of the Federal State Budgetary Research Institution Saratov Federal Scientific Centre of the Russian Academy of Sciences, 410049 Saratov, Russia

**Keywords:** biofilm, exopolymer matrix, Fourier transform infrared spectroscopy, FTIR, plant-growth-promoting rhizobacteria (PGPR), *Azospirillum baldaniorum*

## Abstract

Biofilms represent the main mode of existence of bacteria and play very significant roles in many industrial, medical and agricultural fields. Analysis of biofilms is a challenging task owing to their sophisticated composition, heterogeneity and variability. In this study, biofilms formed by the rhizobacterium *Azospirillum baldaniorum* (strain Sp245), isolated biofilm matrix and its macrocomponents have for the first time been studied in detail, using Fourier transform infrared (FTIR) spectroscopy, with a special emphasis on the methodology. The accompanying novel data of comparative chemical analyses of the biofilm matrix, its fractions and lipopolysaccharide isolated from the outer membrane of the cells of this strain, as well as their electrophoretic analyses (SDS-PAGE) have been found to be in good agreement with the FTIR spectroscopic results.

## 1. Introduction

The diverse field of microbiology has been progressively developing over the last decades, largely owing to the steadily growing applications of a range of modern molecular spectroscopy techniques that provide molecular-level information on complicated microbiological objects and allow for successfully solving various bioanalytical problems, often in situ or in vivo (see, e.g., [1,2,3,4,5,6,7,8,9,10,11,12,13,14,15,16,17]). We would like to emphasize the informativity of very relevant vibrational spectroscopic techniques, including various modifications of infrared (conventional absorption [2,7,11,16], diffuse reflectance [3,6], attenuated total reflectance (ATR) [5,17] and surface-enhanced absorption [15] modes; 2D infrared [8]) and Raman [1,2,13] spectroscopies, as well as their combinations [4,14]; vibrational circular dichroism [12], optical activity [9], etc. Of special notice are microbiological applications of Mössbauer spectroscopy, in both transmission and emission variants [2,10].

Among these techniques, Fourier transform infrared (FTIR) spectroscopy, which has already become a routine tool in material science, yet could not be regarded as routine in the life sciences [4,6,7], and particularly in microbiology [3,5,8,13,14,15,17,18,19]. This situation can in part be explained by the intrinsically sophisticated compositions and structures of diverse microbiological objects, making the processes of sample treatment or preparation for spectroscopic studies, together with treatment of the spectroscopic data and interpreting the results, a challenging task [19]. 

FTIR spectroscopy provides highly informative data and is sensitive not only to the molecular structure of functional groups and ‘backbones’ in bio(macro)molecules, but also to intermolecular interactions (even weak), which are known to play a vital role in biological processes. This sensitivity, on the other hand, also represents “the dark side of the Moon”, consisting in the complication of the resulting spectra owing to the band shifting and overlapping, with a possible appearance of additional bands. Thus, a properly measured FTIR spectrum of an adequately prepared microbiological sample can provide a wealth of structural and compositional information which, however, has to be professionally ‘extracted’ from the spectroscopic data.

The overall chemical composition and structural ‘hierarchy‘ of microbial cells are known to be highly complicated and specific with regard to the genera, species and sometimes even strains. Moreover, the overall cell composition can also rapidly alter depending on the growth phase and/or in response to the changing environments. This complexity is probably the main reason that, despite a great number of studies involving FTIR spectroscopy in microbiology reported so far (see, e.g., [3,5,8,14,15,17,18,19]), the available standardised protocols for FTIR spectroscopic microbiological analyses (including sample preparation) are insufficient, not yet fully generalised and cannot cover all possible cases [19,20,21,22]. Thus, in the review chapter by Ojeda and Dittrich [19], a general overview was presented covering the different sample preparation protocols for infrared spectroscopic analysis of bacterial cells, the basic principles of the technique, procedures for calculating vibrational frequencies based on simple harmonic motions, as well as the advantages and disadvantages of FTIR spectroscopy for the analysis of microorganisms. The earlier protocol-type paper by Martin et al. [20] emphasized the importance of combined spectroscopic and computational analyses of complicated biological specimens and a multivariate approach (principal component analysis with or without linear discriminant analysis). The following large collective work by experts in biospectroscopy [21] presented a broader discussion on similar important points, also discussing difficulties related to determining a consensus on spectral pre-processing and data analysis. They also described a protocol for collecting infrared spectra and images from biological samples which assesses the instrumental options available, appropriate sample preparation, different sampling modes, as well as important advances in spectral data acquisition. The more recent collective paper [22] accentuated the urgent need for repetition and validation of FTIR biospectroscopic analytical methodologies in large-scale studies and across different research groups, which would bring the method closer to clinical and/or industrial implementation. While some of these papers include tissue analyses [20,21], the problems of spectroscopic bioanalysis are evidently common, especially for such supramolecular structures as biofilms. Therefore, such methodology-related studies in the microbiological field still remain highly topical and can be very useful as reference examples in ongoing microbiological research involving this technique.

In our recent reports, several examples of FTIR spectroscopic analyses of biomacromolecular and bacterial cell samples were discussed, with an emphasis on some important methodological and sample preparation effects [23,24,25]. Since biofilms represent the main mode of bacterial existence and play very significant roles in many industrial, medical and agricultural fields (for recent reviews, see, e.g., [26,27,28,29,30,31]), in the present study, the attention is paid to some important methodological aspects of bacterial biofilm analysis using FTIR spectroscopy. It has to be noted that over the last decades, there have been only a couple of thematically limited reviews related to the use of FTIR spectroscopy for studying biofilms. They deal with bacterial biofilm infections involving many clinically relevant microorganisms [32] and consider different spectroscopic techniques (including a ca. 2-page-long part on FTIR spectroscopy) used to characterise microbial biofilms [33].

Thus, the subjects of this work were biofilms formed by the ubiquitous plant-growth-promoting rhizobacterium (PGPR) *Azospirillum baldaniorum*, strain Sp245 (recently reclassified into the above-mentioned separate species from *A. brasilense* [34]). This PGPR species belongs to the genus *Azospirillum*, that has been studied worldwide owing to many beneficial traits and, particularly, agricultural significance of its several phytostimulating species (for most recent reviews, see [35,36,37,38,39,40]). It should be emphasized that studies specially devoted to biofilms formed by *Azospirillum* spp. have been relatively limited (see, e.g., reports [41,42,43,44,45,46,47,48,49,50,51] (and some earlier references cited therein) on various biochemical and physiological properties of biofilms formed by these bacteria, including a report on dual-species biofilms [51]), so this important field for azospirilla is yet to be investigated in more detail. 

As for FTIR spectroscopy, to the best of our knowledge, it has been used only once (in our previous work [52]) for comparatively analysing biofilms of *A. baldaniorum* Sp245 (previously known as *A. brasilense* Sp245) and its mutant strain Sp245.1610 (with alterations in the synthesis of fatty acids, the amount of biomass and relative content of lipopolysaccharide antigens in mature biofilms) formed in the growth medium on ZnSe surfaces. Those analyses emphasized differences in the synthesised amounts of the reserve carbon and energy storage biopolymer material, poly-3-hydroxybutyrate (PHB), which is typical of azospirilla (see [23,24,25] and references therein). In this report, FTIR spectroscopic analyses of *A. baldaniorum* Sp245 biofilms formed on a solid surface or liquid/air interface, as well as the macromolecular biofilm matrix and its main macrocomponents, in comparison with novel data of their chemical and electrophoretic analyses, are reported for the first time from the viewpoint of methodological and compositional aspects.

## 2. Results and Discussion

### 2.1. FTIR Spectroscopic Characterisation of A. baldaniorum Sp245 Biofilms and Their Macrocomponents

#### 2.1.1. Optimisation of the Sample Preparation Procedure for Biofilms of *A. baldaniorum* Sp245 Formed on Solid Surfaces for FTIR Spectroscopic Analysis

In our previous report [25], the effects of drying and grinding of bacterial biomass prepared for FTIR spectroscopic analysis were studied. As is common for this technique, the biomass was washed from the culture medium before drying [25]. Since for the biofilms formed on a solid surface the amount of the harvested biomass can be smaller than that for planktonic cultures and washing steps inevitably lead to its partial loss, in this work, we determined the minimal possible number of washing steps for the biofilm samples.

**Figure 1:**
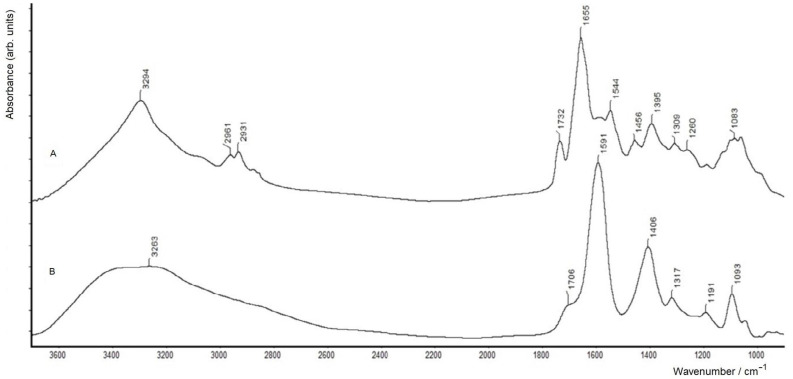
FTIR spectra of (**A**) an *A. baldaniorum* Sp245 biofilm formed on the surface of a ZnSe glass disc (washed one time and dried) and (**B**) a thin dry film of the culture liquid (SMS) measured in the transmission mode on a ZnSe glass disc.

Indeed, it was found that multiple washing (2–3 times) for biofilms formed on a solid surface (ZnSe glass discs), which had been applied before for samples of planktonic bacterial cultures and gave useful results [24,25], led to a considerable loss of biomass from the samples. As a consequence, their FTIR spectroscopic measurements could give inappropriate results, owing to the insufficient amounts of biomass. Therefore, in this case, the use of a single washing step was applied to keep the necessary biofilm material sufficient for measurements. Thus, the biofilm grown on the surface of a ZnSe glass disc (used for FTIR spectroscopic measurements in the transmission mode as shown earlier [25]) was carefully washed one time with physiological saline and dried. Figure 1 compares an FTIR spectrum of such an *A. baldaniorum* Sp245 biofilm, washed one time and dried, with a spectrum of a dry film of the culture medium (see Materials and Methods, Section 3.1).

Figure 1 shows that the overall shape of the FTIR spectrum of the singly washed dry biofilm (spectrum A) is generally typical of samples of bacterial biomass [24,25] and is quite similar to their spectra (positions of the main bands in spectrum A of the biofilm are listed in Table 1 (data for B_ZnSe_)). However, upon careful examination, one can distinguish a weak, but clearly seen contribution from the culture medium components (represented by spectrum B) in the region of its main bands (at least, in the region of its strongest band at 1591 cm^−1^ in spectrum B; see spectrum A). Spectrum B of the dried culture medium is dominated by strong carboxylate vibrations of malate (see Section 3.1), featuring the antisymmetric and symmetric COO^−^ stretching vibrations at 1591 and 1406 cm^−1^, respectively. Therefore, in this case (with a single washing step for a biofilm sample with a limited amount of biomass, formed on a solid surface), quantitative analyses of FTIR spectra have to be undertaken with caution, considering possible contributions from the remaining culture medium components in the dried biomass. Comparative analyses of particular biomacromolecular components in a biofilm, in this case, may be performed within spectroscopic regions which do not coincide with those with the maximum absorption of the culture medium (e.g., see Figure 1, spectrum B). A better strategy would, thus, consist in obtaining larger amounts of biofilm biomass, to ensure the possibility of applying two or three washing steps.

#### 2.1.2. FTIR Spectroscopic Analyses of *A. baldaniorum* Sp245 Biofilm Formed at the Air–Liquid Interface, the Biofilm Matrix and Its Macrocomponents

**Figure 2:**
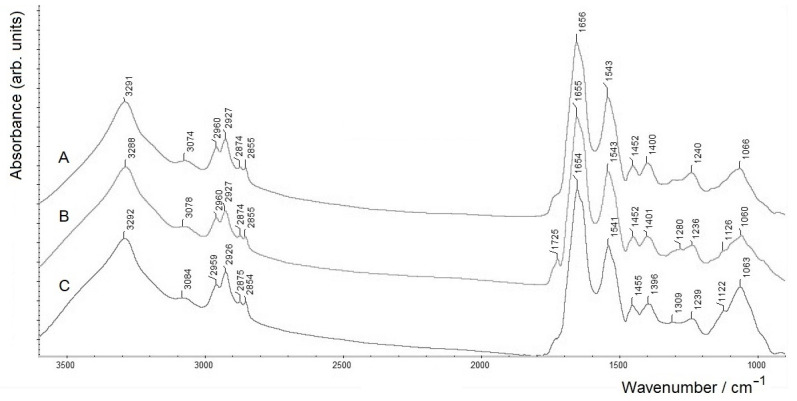
FTIR spectra of (**A**) an *A. baldaniorum* Sp245 biofilm formed at the air–liquid interface (washed triply and dried), (**B**) a thin, dry, film of bacterial cells separated from the biofilm and (**C**) its matrix (BM), measured in the transmission mode on a ZnSe glass disc.

In order to perform a more accurate analysis by FTIR spectroscopy, an *A. baldaniorum* Sp245 biofilm was grown for 5 days at the air–liquid interface (where more biofilm biomass could be obtained). The mature biofilm was separated from the medium containing suspended planktonic cells; for FTIR spectroscopic measurements, it was washed triply with physiological saline and dried (sample B_A/L_). Part of this biofilm was used to separate its bacterial cells (sample Cells(B_A/L_)) and the crude biofilm matrix (sample BM; see Section 2.2). These three samples were further compared using FTIR spectroscopy (Figure 2).

All three FTIR spectra in Figure 2 have very similar shapes, characteristic of bacterial samples [24,25]. Note that spectrum A of the biofilm in Figure 2, which was washed triply, shows no signs of the components of the growth medium (i.e., an increased absorption around ~1600 and ~1400 cm^−1^, as seen in spectrum A in Figure 1 for the biofilm washed only once). As can also be seen, the spectrum of the biofilm (spectrum A) only slightly differs from the measured spectra of isolated cells (spectrum B) and BM (spectrum C), which indicates similar ratios of macrocomponents in their compositions (see also Table 1; samples B_A/L_, Cells(B_A/L_) and BM, respectively). Slight differences in the measured FTIR spectra can be observed only in the region of the bands at ~1730 cm^−1^, corresponding to the stretching vibrations of the ester C=O functional group (a spectroscopic marker of the biopolyester PHB in azospirilla [24]), which in the absence of PHB accumulation by cells could be noticeable as a very weak band (or rather a shoulder) in cellular lipopolysaccharides (LPS) and phospholipids (see, e.g., [24]). In spectrum B of individual cells, this weak ν(C=O) band has the highest intensity compared to those in the spectra of the biofilm and its matrix. The somewhat greater intensity of the ν(C=O) band for the separated cells (compared to that for the biofilm) can be associated with the removal of the matrix from the biofilm sample, in the spectrum of which, as can be seen (spectrum C), the ν(C=O) band is minimal in intensity. 

Comparing the FTIR spectra of the biofilms in Figure 1 (spectrum A) and Figure 2 (spectrum A), one can see that the ν(C=O) band in the former (at 1732 cm^−1^) is relatively stronger than that in the latter (featured by a shoulder). While this band reflects the rate of PHB accumulation and could, thus, generally depend on the biofilm growth conditions (which slightly differ for these samples; see Section 3.1), the main reason may be that for the biofilm grown on ZnSe, the concentration of the bound nitrogen source (NH_4_Cl) in the growth medium was 0.5 g·L^−1^, while for the biofilm grown at the air–liquid interface, it was twice as high (1.0 g·L^−1^; see Section 3.1). Note that for bacteria of the genus *Azospirillum*, a deficiency of bound nitrogen in the growth medium (i.e., an increased C:N ratio of bioavailable nutrients) is known to be one of the factors inducing PHB biosynthesis and its accumulation in cells (see, e.g., [24] and references reported therein).

It might also be noted that for the isolated BM (spectrum C), the absorption in the polysaccharide-related region (~1200–950 cm^−1^) is slightly larger than in spectra A and B, while in all three spectra the protein-related bands (amide I at ~1655 cm^−1^ and amide II at ~1543 cm^−1^ [24,25]) evidently dominate.

**Figure 3:**
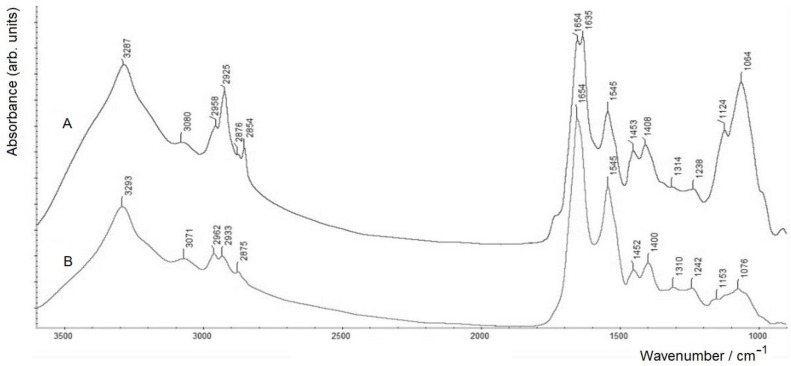
FTIR spectra of *A. baldaniorum* Sp245 biofilm matrix components BM1 (**A**) and BM2 (**B**) obtained by separating the crude biofilm matrix (sample BM) on a column with a Sepharose CL-6B carrier (measured in the transmission mode on a ZnSe glass disc).

Separation of the BM sample on a column with a Sepharose CL-6B carrier allowed two fractions with different molecular weights (BM1 and BM2) to be obtained (see Section 2.2). The FTIR spectra of these samples were also found to differ markedly from each other (Figure 3).

The first striking difference can be seen in the spectroscopic region typically related to polysaccharides (~1200–950 cm^−1^). The FTIR spectrum of sample BM1 (spectrum A) is characterised by a much more intense band of the polysaccharide component, as compared to that in spectrum B of sample BM2. The other accompanying differences consist in the presence of a shoulder characteristic of lipids (ester ν(C=O) vibrations at ca. 1735 cm^−1^) in spectrum A of BM1 (which is significantly weaker in spectrum B of BM2), as well as noticeably enhanced regions related to the stretching vibrations of –CH_3_ and –CH_2_– groups within 3000–2800 cm^−1^ and the corresponding bending vibrations at 1453 cm^−1^ in spectrum A (see also Table 1). Note that in spectrum A of BM1, both of the ν(C–H) vibrations of methylene groups (at 2925 and 2854 cm^−1^) are noticeably stronger than those of the terminal –CH_3_ groups (the proportion of the latter is evidently much lower in long aliphatic, especially alkanoic, chains). Altogether these spectroscopic differences indicate that in BM1, the increased polysaccharide content is related to the LPS (see also Section 2.2), with their typical aliphatic chains of fatty acid residues.

Another striking feature in spectrum A of sample BM1 is in the region of amide I (protein component), where there are two maxima corresponding to the different secondary structure components: α-helices (at 1654 cm^−1^) and β-sheets (at 1635 cm^−1^; see the corresponding discussions on the sensitivity of the amide I region in FTIR spectra to the secondary structure of proteins, e.g., in [25,53,54], etc.). For BM2, in the region of the amide I band in spectrum B, there is a single maximum at 1654 cm^−1^, which corresponds to the predominant content of the secondary structure of the protein components in the form of an α-helix.

**Figure 4:**
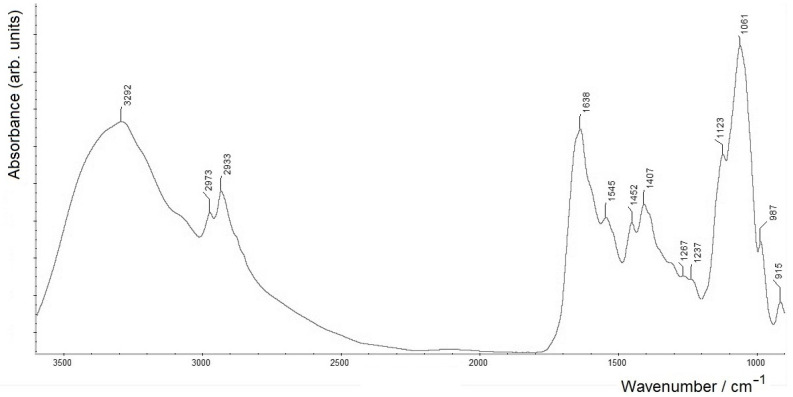
FTIR spectrum of the polysaccharide fraction (sample BM3) obtained from *A. baldaniorum* Sp245 biofilm matrix by mild acidic hydrolysis (measured in the transmission mode on a ZnSe glass disc).

In an attempt to isolate the polysaccharide components of the BM sample, mild acid hydrolysis of the BM preparation, with 2% acetic acid at 100 °C (4 h), was performed (yielding sample BM3). An FTIR spectrum of this sample (Figure 4) shows all the typical intense bands of the polysaccharide component (see also Table 1). 

Interestingly, along with these bands, the FTIR spectrum also shows clearly resolved bands within the protein-related region (amide I and amide II at 1638 and 1545 cm^−1^, respectively) with a somewhat distorted ratio. It is noteworthy that similar amide I and amide II bands in infrared spectra could be related to amide bonds in the absence of proteins. In this case, while the results of electrophoretic analysis of sample BM3 showed its full similarity with the LPS from the outer cell membrane of this bacterium and the absence of protein components, these amide I and amide II bands can evidently be assigned to amido-bonded fatty acid residues (see further discussion in Section 2.2). Note that in Figure 4, a relatively more intense ν_s_(COO^−^) band at 1407 cm^–1^ (see Table 1), as compared to those in Figure 2 and Figure 3, matches well with a noticeable shoulder at ~1600 cm^−1^. The latter features the corresponding antisymmetric vibration mode, ν_s_(COO^−^), with both vibrations evidently corresponding to the carboxylic groups in polysaccharide moieties of LPS (cf. also the related positions of the carboxylate bands in Figure 1, spectrum B, and their signs in spectrum A).

### 2.2. Chemical Characterisation of A. baldaniorum Sp245 Biofilm Matrix Components

To illustrate the FTIR spectroscopic data discussed above on the macro-composition of *A. baldaniorum* Sp245 BM and its fractions (BM1, BM2 and BM3; see Figure 2, Figure 3 and Figure 4 and Table 1), a chemical characterisation of these samples was performed. For comparison, a sample of the LPS isolated from the outer membrane of *A. baldaniorum* Sp245 cells was also analysed (Table 2).

It has to be noted that the formation of biofilms is a complex, strictly regulated biological process, as a result of which the bacterial community, united by complex intercellular connections, switches to a qualitatively different way of functioning [27,55,56,57,58]. It is known that the process of initiation of biofilm formation and different stages of its development are accompanied by differential expression of bacterial genes [56,59]. The formation of a mature biofilm is assessed by reaching its maximum thickness (in the case of sample B_A/L_, it was ~82 ± 7 μm; see Section 3.1), which is further maintained at a constant level for a long time. This stage is characterised by the lowest variability of the matrix composition, especially under constant cultivation conditions, and is finally followed by the stage of dispersion and degradation [59].

**Table 2:** Positions of the maxima of typical absorption bands (in cm^−1^) in FTIR spectra of dried samples of biofilms formed by *A. baldaniorum* Sp245 at the ZnSe glass surface (B_ZnSe_; see Figure 1A) or at the air–liquid interface (B_A/L_; see Figure 2A) and its isolated cells (Cells(B_A/L_); see Figure 2B), biofilm matrix (BM; see Figure 2C) and its fractions: BM1 (see Figure 3A), BM2 (see Figure 3B) and BM3 (see Figure 4), and their assignments ^1^ [23,24,25,52,53,54,55,58].

Assignment (Functional Groups)	B_ZnSe_	B_A/L_	Cells (B_A/L_)	BM	BM1	BM2	BM3
O–H; N–H (amide A in proteins), ν	3294	3291	3288	3292	3287	3293	3292
C–H in methyl groups –CH_3_ (ν_as_)	2961	2960	2960	2959	2958	2962	2973
C–H in methylene groups >CH_2_ (ν_as_)	2931	2927	2927	2926	2925	2933	2933
C–H in methyl groups –CH_3_ (ν_s_)	~2875w	2874w	2874w	2875w	2876w	2875	~2876sh
C–H in methylene groups >CH_2_ (ν_s_)	~2855w	2855w	2855w	2854w	2854	~2854sh,w	~2854sh
Ester C=O, ν (phospholipids; PHB)	1732	~1730sh	1725	~1730sh,w	~1735sh	~1735sh,w	~1730sh,w
Amide I (proteins; amide bonds)	1655	1656	1656	1654	1654;1635	1654	1638
Amide II (proteins; amide bonds)	1544	1543	1543	1541	1545	1545	1545
–CH_2_– and –CH_3_, δ (in proteins, lipids, sugars, etc.)	1456	1452	1452	1455	1453	1452	1452
COO^−^, ν_s_ (in amino acid side chains and carboxylated polysaccharides) ^2^	1395	1400	1401	1396	1408	1400	1407
C–O–C/C–C–O, ν (in esters; PHB, etc.)	1309	~1309w	1280	1309	1314	1310	1267
C–O–C (esters)/amide III/O–P=O, ν_as_	1269	1240	1236	1239	1238	1242	1237
C–O, C–C, C–OH, ν; C–O–H, C–O–C, δ (in polysaccharides)	1129sh, ~1190w	~1125sh	1126	1122	1124s	1153	1123s
O–P=O, ν_s_; C–O, C–OH	1083	1066	1060	1063	1064s	1076	1061s

^1^ Notations for vibration modes: ν—stretching; ν_s_—symmetric stretching; ν_as_—antisymmetric stretching; δ—bending; sh—shoulder; w—weak; s—strong. ^2^ The corresponding antisymmetric stretching vibrations (ν_as_ of COO^−^, commonly of higher intensities than ν_s_) may have variable positions (observed usually around ~1650–1580 cm^−1^); in a microbial biomass, they are commonly masked by significantly more intensive amide I/II bands of cellular proteins.

The exopolymer matrix is extremely important for the survival and living of cells in the biofilm, namely for the maintenance of its three-dimensional architecture, adhesion to various surfaces, the role of a protective barrier and a source of nutrients, etc. [56]. The BM components of various bacteria comprise exopolysaccharides, extracellular proteins, lipids and nucleic acids, the ratio of which can vary in a wide range [57]. For strain *A. baldaniorum* Sp245, it was shown that exopolysaccharide is an extracellular form of LPS with an identical structure of the polysaccharide part, linear D-rhamnan [60].

The obtained crude BM preparation was featured by a high protein content (see Table 2). It also contained carbohydrate components, phosphoric acid residues, as well as 3-deoxy-D-manno-octulosonic acid (Kdo), the marker component of the LPS of Gram-negative bacteria.

Analysis of fatty acids (FAs) in the BM composition by gas chromatography (GC) showed the predominance of octadecenoic (62% of total FA methyl esters, FAME), hexadecenoic (11%) and hexadecanoic (10%) acids. The predominance of these fatty acids and their ratio are a chemotaxonomic criterion for bacteria of the genus *Azospirillum*. Lipids and biosurfactants are known to play an important role in the formation of biofilms, affecting the surface tension at the air–liquid interface [56]. A high content of unsaturated FAs can provide the biofilm architecture with the necessary “fluidity”. The presence of 3-hydroxytetradecanoic and 3-hydroxyhexadecanoic acids (with their total content about 10%), which are markers for lipopolysaccharides of azospirilla, was also shown [61,62].

**Figure 5:**
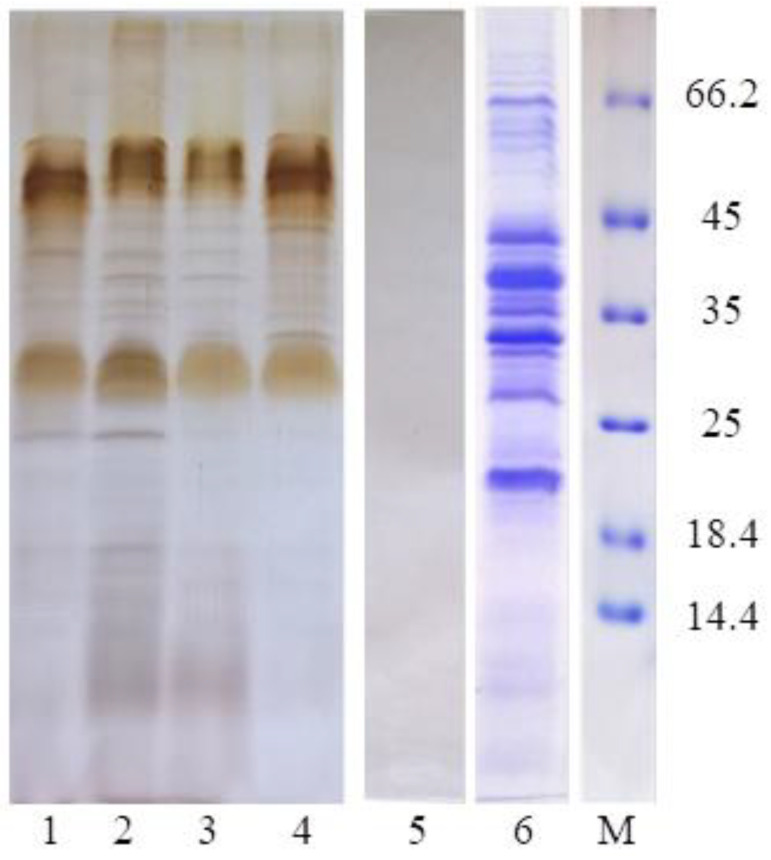
SDS–PAGE, followed by staining with AgNO_3_-based dye (lanes 1–4) and Coomassie brilliant blue (lanes 5, 6, M) of the following samples: LPS (lane 1), deproteinated BM (lane 2) and native crude BM (lanes 3, 6), as well as BM3 (lanes 4, 5). Lane M represents protein markers (with their corresponding molecular masses in kDa).

Analysis by sodium dodecyl sulfate–polyacrylamide gel electrophoresis (SDS-PAGE) showed a predominance of protein components in the BM crude extract in the range ~20–100 kDa (Figure 5). The similarity of the electrophoretic profile of LPS, previously isolated from cells of the studied strain, with those of native and deproteinated BM (when stained with silver nitrate after periodate oxidation) showed that the carbohydrate component of the matrix is represented by the LPS.

**Figure 6:**
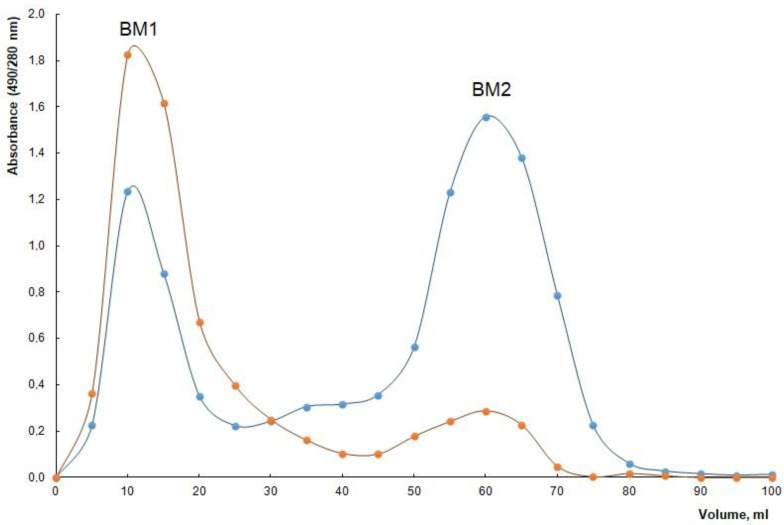
Elution profiles of *A. baldaniorum* Sp245 biofilm matrix on a Sepharose CL-6B column (showing fractions BM1 and BM2). The carbohydrate content was detected by the phenol–sulfuric acid method (brown line) and the protein content was detected by absorbance at 280 nm (blue line).

When separating the matrix components by gel filtration, two fractions (BM1 and BM2) were obtained in a ratio of ~2.5:1, differing in their composition (Table 2, Figure 6). Both fractions evidently contain protein and carbohydrate components, but in different proportions. As follows from Figure 6, each of the isolated fractions (BM1 and BM2) of the matrix, in line with the analyses of their compositions (see Table 2) and chromatographic separation data (broadened peaks in Figure 6), is represented by a complex of proteins and the LPS. The heterogeneity of these fractions, due to their multi-component nature, does not allow the molecular weights of the individual substances included in their composition to be accurately characterised. It can only be assumed, basing on the fractional range of the chromatographic carrier, that the ranges of the mass distribution are approximately 80–40 kDa for BM1 and 35–20 kDa for BM2.

Further, in order to elucidate the nature of the carbohydrate component of the *A. baldaniorum* Sp245 BM, the crude BM fraction was subjected to mild acidic hydrolysis with 2% acetic acid at 100 °C, which led to the denaturation of all proteins that were precipitated by centrifugation. At the same time, the BM3 fraction showed a high content of carbohydrates, Kdo and phosphate (see Table 2), and its electrophoretic profile completely coincided with that of the LPS isolated from the outer membrane of *A. baldaniorum* Sp245 cells (Figure 5). The presence of 3-hydroxylated fatty acids was also shown in BM3.

According to the results of chemical analysis and electrophoresis data, there were no protein components in BM3. Analysis of the monosaccharide composition revealed the presence of a single sugar, D-rhamnose, which had previously been found in the composition of the LPS and capsular polysaccharide (CPS) of the studied strain [60,63].

It should be noted that LPS and exopolysaccharide of *A. baldaniorum* Sp245 were found to contain phosphoric acid residues, the content of which increased together with the duration of bacterial cultivation [60]. In the BM3 fraction of *A. baldaniorum* Sp245, the content of phosphoric acid residues was 2.3% (see Table 2). The canonical structure of lipid A of the members of the family *Enterobacteriaceae* consists of a β1,6-linked glucosamine dimer backbone and phosphate groups at the 1 and 4’ positions of the backbone [64].

When studying the structure of lipid A for two *Azospirillum* strains, *A. lipoferum* SpBr17 [65] and *A. rugosum* DSM-19657 [62], their fundamental difference was shown, consisting in the absence of phosphorylation and the presence of a trisaccharide backbone of the following structure: GlcpN(1→6)GlcpN(1↔1)GalpA, in which the GlcpN residues are acylated with 3-hydroxyhexadecanoic acid at positions 2 and 2′ and with 3-hydroxytetradecanoic acid at positions 3 and 3′, while the residues of secondary acids esterify 3-hydroxyhexadecanoic acid at position 2′. In [62], it was shown basing on the data of chemical analysis and MALDI mass spectra, that lipid A from *A. rugosum* DSM-19657 is a mixture of penta-, tetra- and triacyl types which differ from each other in the number of residues of the primary O-linked 3-hydroxytetradecanoic acid and the composition of secondary acids, while amide-bound 3-hydroxyhexadecanoic acid was found in all forms of lipid A.

Given the conservatism of the lipid A structure within the bacterial genus, we can assume a similar structure of lipid A in the studied strain and the possible phosphorylation of the Kdo residue that links the core and lipid A moieties. As a result of the latter, phosphate groups can shield the acid-labile bond, which, in turn, hinders the hydrolysis of lipopolysaccharide [61]. The presence of non-degraded lipopolysaccharide in the BM3 preparation explains the presence of amide I and amide II signals in its FTIR spectrum, as well as signals from aliphatic FA groups, and the microheterogeneity of lipid A, noted above, can be the cause of rather strong amide I and amide II signals (see Figure 4).

Thus, it can be summarised that the results of chemical and electrophoretic analyses of the *A. baldaniorum* Sp245 BM and its fractions (see Table 2 and Figure 5 and Figure 6) are in good agreement with the data of FTIR spectroscopic analyses of their samples (see Section 2.1.2, Figure 2, Figure 3 and Figure 4 and Table 1). These detailed analyses have been performed for the first time for *A. baldaniorum* Sp245 biofilms formed at the air–liquid interface.

## 3. Materials and Methods

### 3.1. Cultivation of A. baldaniorum Sp245 and Growth of Biofilms

Wild-type strain *Azospirillum baldaniorum* Sp245 [34] (previously known as *Azospirillum brasilense* Sp245 [66]) was obtained from the Collection of Rhizosphere Microorganisms (WDCM 1021) maintained at the Institute of Biochemistry and Physiology of Plants and Microorganisms–Subdivision of the Federal State Budgetary Research Institution Saratov Federal Scientific Centre of the Russian Academy of Sciences, Saratov, Russia [67] (http://collection.ibppm.ru/catalogue/azospirillum/azospirillum-brasilense/) (accessed on 17 February 2023).

Bacterial pre-cultures were cultivated in the air for 18 h at 30 °C in a liquid modified malate salt medium (MSM) [68], which contained the following substances (g·L^−1^): K_2_HPO_4_, 3.0; KH_2_PO_4_, 2.0; NH_4_Cl, 0.5 (for biofilms on ZnSe discs; see Section 2.1.1) or 1.0 (for biofilms at the air–liquid interface; see Section 2.1.2); NaCl, 0.1; FeSO_4_·7H_2_O, 0.02 (added as chelate with nitrilotriacetic acid, 0.056); CaCl_2_, 0.02; MgSO_4_·7H_2_O, 0.2; Na_2_MoO_4_·2H_2_O, 0.002; sodium malate, 5.0 (obtained by mixing 3.76 g of malic acid with 2.24 g NaOH per litre), pH 6.8–7.0, in Erlenmeyer flasks (250 mL; with 100 mL of the medium).

Biofilms of *A. baldaniorum* Sp245 were grown either on the surface of ZnSe glass discs (CVD-ZnSe, “R’AIN Optics”, Dzerzhinsk, Russia; ø 2.5 cm, thickness 0.2 cm) placed on the bottom of a Petri dish (ø 4 cm, with 3 mL of the inoculated MSM) in a thermostat at 28 °C for 6 d (see Section 2.1.1), or at the air–liquid interface (see Section 2.1.2) in Erlenmeyer flasks (1 L, with 700 mL of the inoculated medium) at 27 °C for 5 d, without stirring. When cultivated on a liquid nutrient medium under stationary conditions, after 120 h of growth, *A. baldaniorum* Sp245 formed mature biofilms at the air–liquid interface, with the maximum thickness of 82.4 ± 7.4 μm. The biofilm thickness was determined by phase contrast microscopy on a Leica LMD7000 laser dissector (Leica Microsystems, Wetzlar, Germany). The preparation of specimens for microscopy and analysis of the results were performed according to [42]. Measurements were made for at least three biofilm samples during each cultivation; for each biofilm sample the thickness was determined at 10 to 20 randomly selected points.

Mature biofilms (520 mg), formed at the air–liquid interface, were separated by filtration from the suspension culture through a coarse nylon filter, washed with a 0.1 M phosphate buffer (pH 7.2) and dried at 60 °C for 1 day.

### 3.2. Separation of Macrocomponents of A. baldaniorum Sp245 Biofilm

After separation from the planktonic culture, the biofilm formed at the air–liquid interface was suspended in 0.1 M phosphate buffer (pH 7.2) and sonicated twice (37 kHz, 40 °C, 30 min). Bacterial cells from the biofilm (280 mg) were pelleted by centrifugation (3000× *g*, 40 min), resuspended twice in acetone and dried in air at room temperature. The supernatant containing crude BM was dialysed against distilled water for 2 d, evaporated at 40 °C under reduced pressure (Laborota 4000; Heidolph, Schwabach, Germany) and lyophilised in a Benchtop 2K freeze dryer (VirTis, Gardiner, NY, USA).

The crude BM (20 mg) was redissolved in water and fractionated by gel permeation chromatography (GPC) on a Sepharose CL-6B column (2.5 × 46 cm; GE Healthcare, Chicago, IL, USA), by using 0.025 M NH_4_HCO_3_ (pH 8.3) as the eluent, and monitoring with a differential refractometer (2142; LKB, Bromma, Sweden). Both fractions, BM1 (10 mg) and BM2 (4 mg), were further assayed for biopolymer composition.

The polysaccharide fraction (BM3) was obtained by a degradation of the crude BM (100 mg) with aqueous 2% acetic acid (100 °C, 4 h), followed by GPC on a column of Sephadex G 50 (S) (56 × 2.6 cm, GE Healthcare, Chicago, IL, USA), using 0.05 M pyridinium acetate (pH 4.5) as the eluent, and monitoring with a differential refractometer (2142; LKB, Bromma, Sweden). The yield of BM3 was 15% of the crude BM. 

### 3.3. Colorimetric Assay

Total sugar concentrations in all BM-related samples were determined by the phenol–sulfuric acid method [69]. Briefly, 2 mg from each dried BM fraction were dissolved in 10 mL of deionised water, then 400 μL of the dissolved sample and 400 μL of 5% phenol solutions (w/v) were mixed with 2 mL of 95% H_2_SO_4_, strongly vortexed and left at room temperature for 20 min. The absorbance was measured at 490 nm, and all measurements were completed in triplicate. d-(+)-Glucose was used as a standard for a calibration curve. 

The protein content was determined using the Bradford method [70]: 1 mL of the prepared solution of a BM-related sample (0.2 mg/mL) was mixed with 1 mL of a Coomassie brilliant blue G-250 solution, agitated vigorously and left for 10 min. The absorbance was measured at 595 nm. BSA was utilised as a standard for a calibration curve.

The content of phosphate was determined by the method of Berenblum and Chain [71]. 

The content of 3-deoxy-d-manno-2-octulosonic acid (Kdo) in the BM-related samples was determined after treatment of a 2-mg sample with 0.2 N H_2_SO_4_ (1 mL, at 100 °C for 30 min) to release Kdo, followed by its reaction with 0.04 M periodic acid in 0.125 N H_2_SO_4_ (0.25 mL at 20 °C for 20 min), 2.6% sodium arsenite in 0.5 N HCl (until decoloration), and 0.6% aqueous solution of thiobarbituric acid (0.5 mL at 100 °C for 10 min). The red chromophore formed was kept in solution at room temperature by adding dimethyl sulfoxide to the reaction mixture [72].

### 3.4. Analysis of Monosaccharide and Fatty Acid Composition

The monosaccharide composition of BM-related samples was achieved as alditol acetates [73] by gas liquid chromatography (GLC) analysis. Briefly, 1 mg of a sample was hydrolysed by 1 mL of 2 M trifluoroacetic acid (TFA) at 120 °C for 2 h. Methanol was added to the system, followed by evaporation to dryness in order to remove TFA, and the obtained substance became a hydrolysate, followed by its reaction with a solution of 0.25 M NaBH_4_ in 1 M ammonia (0.5 mL at 20 °C for 1 h). After neutralisation with 10% acetic acid in methanol, followed by evaporation, 0.5 mL of pyridine and 0.5 mL of acetic anhydride were added to the sample (kept at 100 °C for 45 min). All standard sugars (glucose, xylose, arabinose, mannose, rhamnose, fucose, galactose) were converted to their acetylated derivatives, according to the methods described above. The alditol acetates were analysed on a DB-5 capillary column (30 m × 0.32 nm, 0.25 μm) (Agilent, Santa Clara, CA, USA), by using a GC-2010 chromatograph (Shimadzu, Kyoto, Japan). The injection volume was 1 μL; the split ratio was 10:1; the carrier gas was ultra-pure nitrogen; and the temperature of the detector was 270 °C. The temperature gradient was 160 °C (1 min) to 250 °C at 7 °C·min^−1^.

The fatty acids of the BM-related samples were determined as fatty acid methyl esters (FAMEs) [74] using a GC-2010 instrument (Shimadzu, Kyoto, Japan) equipped with a DB-5 capillary column (Agilent, Santa Clara, CA, USA). The temperature gradient was 130 °C (3 min) to 250 °C at 4 °C·min^−1^. The retention times of the analysed peaks in the samples were compared with those of a standard Bacterial Acid Methyl Ester (BAME) mix (Sigma–Aldrich, St. Louis, MO, USA).

### 3.5. SDS–Polyacrylamide Gel Electrophoresis

The BM-related samples were subjected to electrophoresis in 13.5% SDS–polyacrylamide gel [75]. The components were visualised by staining the gel with a silver-nitrate-based dye (for detecting lipopolysaccharides) [76] and Coomassie brilliant blue R-250 (for detecting proteins) [77]. A Thermo Scientific electrophoresis calibration kit for determining the molecular weight of proteins was reconstituted with an SDS sample diluter. The resulting marker solution, containing BSA (66.2 kDa), ovalbumin (45 kDa), lactate dehydrogenase (35 kDa), REase Bsp98I (25 kDa), β-lactoglobulin (18.4 kDa), and lysozyme (14.4 kDa), was subjected to SDS-PAGE.

To prepare a deproteinated BM sample (see Figure 5, lane 2), for protein digestion, 25 μg of proteinase K from *Tritirachium album* (Sigma-Aldrich, St. Louis, MO, USA) solubilised in 10 μL of the lysing buffer [75] was added to 1 mL of the BM solution (1 mg·mL^−1^) and incubated at 60°C for 60 min.

### 3.6. FTIR Spectroscopic Measurements

All FTIR spectroscopic measurements were performed in the transmission mode, using thin films of samples on ZnSe glass discs (CVD-ZnSe, “R’AIN Optics”, Dzerzhinsk, Russia; ø 2.5 cm, thickness 0.2 cm). 

For FTIR spectroscopic analyses of the *A. baldaniorum* Sp245 biofilm formed directly on the surface of a ZnSe glass disc (see Section 3.1), the prepared biofilm on a ZnSe disc was carefully washed once with physiological saline and dried at 45 °C up to a constant weight. 

For FTIR spectroscopic analyses of all other dry samples (already washed triply before drying), a few mg of the sample were first resuspended in a small volume of Milli-Q water. Then, the resulting aqueous suspension (about 30–70 μL) was placed as a thin film on a clean flat ZnSe disc and dried at 45 °C. Other methodological details of sample preparation for FTIR spectroscopic measurements were discussed earlier [25].

## 4. Conclusions

For the ubiquitous rhizobacterium *Azospirillum baldaniorum* Sp245, widely studied worldwide for its phytostimulation capabilities and agricultural importance [35,37,38], biofilms formed on the solid surface and at the air–liquid interface, and the biofilm matrix components have been investigated in detail for the first time using FTIR spectroscopy, an informative modern technique, together with comparative chemical and electrophoretic analyses. It has been shown that FTIR spectroscopic data on the overall biomacromolecular composition of the biofilm and its exopolymer matrix-related samples under study are in good agreement with their chemical and SDS-PAGE analyses.

## Figures and Tables

**Table 1:** The chemical composition (contents of components in wt.%) of the biofilm matrix (BM), its fractions (BM1, BM2 and BM3), as well as of the LPS from *A. baldaniorum* Sp245.

Components	LPS	BM	BM1	BM2	BM3
Total sugars	55.4 ± 4.5	9.1 ± 1.0	42.1 ± 3.4	10.4 ± 1.8	62.3 ± 4.8
Proteins	–	67.4 ± 0.4	18.7 ± 1.5	53.2 ± 3.7	–
Kdo	2.6 ± 0.2	0.3 ± 0.1	0.9 ± 0.1	0.3 ± 0.1	1.5 ± 0.1
Phosphate	1.4 ± 0.2	1.2 ± 0.1	0.9 ± 0.1	0.8 ± 0.1	2.3 ± 0.3

## Data Availability

The data presented in this study are contained within the article.

## Sample Availability

Samples of the compounds used in the experiments are available from the authors upon request.
